# The union of narrative and executive function: different but complementary

**DOI:** 10.3389/fpsyg.2014.00469

**Published:** 2014-05-20

**Authors:** Margaret Friend, Raven Phoenix Bates

**Affiliations:** Department of Psychology, San Diego State UniversitySan Diego, CA, USA

**Keywords:** executive function, narrative, attention, inhibition, preschool children

## Abstract

Oral narrative production develops dramatically from 3 to 5 years of age, and is a key factor in a child's ability to communicate about the world. Concomitant with this are developments in executive function (EF). For example, executive attention and behavioral inhibition show marked development beginning around 4 years of age. Both EF and oral narrative abilities have important implications for academic success, but the relationship between them is not well understood. The present paper utilizes a cross-lagged design to assess convergent and predictive relations between EF and narrative ability. As a collateral measure, we collected a Language Sample during 10 min of free play. Language Sample did not share significant variance with Narrative Production, thus general language growth from Wave 1 to Wave 2 cannot account for the predictive relations between EF and Narrative. Our findings suggest that although EF and Narrative ability appear independent at each Wave, they nevertheless support each other over developmental time. Specifically, the ability to maintain focus at 4 years supports subsequent narrative ability and narrative ability at 4 years supports subsequent facility and speed in learning and implementing new rules.

## Introduction

Storytelling is integral to human culture: the ability to express a story using pictures and relate it to life is the essence of creating shared meaning. Oral narrative production develops dramatically from 3 to 5 years of age. Concomitant with this development are developments in executive function. For example, executive attention and behavioral inhibition show marked development beginning around 4 years of age. Both executive function and oral narrative abilities have important implications for academic success, but the relationship between them is not well understood.

One form of oral narrative is emergent reading, which occurs when children tell a story using a picture book for support (Sulzby, 1985; Valencia and Sulzby, 1991). Curenton and Justice (2004) found significant increases in the use of conjunctions and verbs in the narratives of preschoolers from 3 to 5 years of age. Story grammar also undergoes maturation during this.

Although children as young as 3 can between past and present tense, they rarely use past tense when telling a story. Tense marking improves along with the use of verbs and conjunctions by age 5 and this contributes to the ability to convey action and organize events in a coherent sequence (Berman and Slobin, 1994). In addition, Nicolopoulou and Richner (2007) found that at age 3 children often focus on physical aspects of characters whereas at age 4, character descriptions include some goal-related action and by age 5, children express a more complex representation of characters in their story telling (Nicolopoulou and Richner, 2007).

Narratives are a product of increasing linguistic sophistication over the preschool period (Kaderavek and Sulzby, 2000) and there is a complex relation between early narratives, language proficiency, and theory of mind (ToM). In a classic paper, Astington and Jenkins (1999) showed that the relation between language and ToM is unidirectional: early language predicts later ToM but early ToM does not predict later language. Charman and Shmueli-Goetz (1998) confirmed a strong relation between language and ToM but found a more limited relation between ToM and narrative: ToM was associated with referential strategy but not with mental state terms, length, complexity, or story structure. Recent work supports this circumscribed view of the relation between ToM and narrative. For example, Fernández (2013) found that ToM explained a small but significant portion of the variance in pragmatic language in children's narratives beyond variance explained by gender and language proficiency. Similarly Ketelaars et al. (2012) found that false belief understanding explained 7% of the variance in the narrative productivity (number of grammatical units, clauses, and MLU) beyond variance explained by language but did not account for variance in story organization or cohesion. This emphasizes the importance of selecting an approach to coding that focuses on the aspects of narrative under investigation. In the present research, we are particularly interested in aspects of narrative production that are likely to be associated with executive function.

Cobo-Lewis et al. (2002) developed a narrative complexity scale to assess narrative construction across languages in bilingual acquisition. This scale captures several aspects of narrative structure that are particularly likely to support and be supported by the development of executive function: memory for story elements, sequencing, demarcating the story with a clear beginning, middle, and end, and using complex syntax. This scale distinguished monolinguals from bilinguals on linguistic elements but not on memory, sequencing, and structure suggesting that these components of narration are not confounded with language proficiency in typically developing children. Using a similar approach focusing on thematic aspects of children's narratives, Ilgaz and Aksu-Koç (2005) found clear improvement in structure from 3 to 5 years of age.

A review of the literature by Mar (2004) found evidence for a network of frontal, temporal, and cingulate areas supporting story comprehension and production. Narrative production and comprehension require substantial organizational skill and are particularly dependent on frontal cortical activation. Troiani et al. (2008) found support for this thesis in a magnetic imaging study of young adults narrating the children's picture story, “Frog, Where Are You” (Mayer, 1969). Peak activations were obtained bilaterally in the inferior frontal cortex as well as the temporal-parietal region and visual association cortex. Troiani et al. concluded that the bilateral frontal activation reflected the top-down organization that is necessary to construct an extended narrative. However the results also suggest a larger network that supports memory for story components, inferential meaning, and story organization.

Concomitant with the emergence of narrative ability, goal-directed action improves dramatically. The psychological processes underlying goal-directed action are referred to collectively as executive function (Zelazo et al., 2003) and there is consensus that substantial changes in executive function occur between 3 and 6 years of age (Carlson, 2003, 2005; Zelazo et al., 2003; Bunge and Zelazo, 2006; Crone et al., 2006; Garon et al., 2008; Moriguchi and Hiraki, 2009; Diamond, 2013). Executive attention, behavioral inhibition, and working memory are foundational higher-level processes that develop in early childhood (Best and Miller, 2010) although other recent work characterizes this triumvirate as set shifting (the ability to shift between rule sets), inhibition, and working memory (Miyake et al., 2000; Garon et al., 2008).

There is substantial theoretical overlap between these processes and shared variance in the tasks that tap them (Stelzer et al., 2014). For example, Best and Miller (2010) place the well- researched Dimensional Change Card Sort (DCCS) task squarely in the domain of complex behavioral inhibition whereas Garon et al. (2008) classify it as a set-shifting task. Further, inhibition tasks often place demands on working memory such that inhibition and working memory are not fully dissociable. Similarly, a recent study of the factor structure of executive function suggests that, in early childhood, set shifting and inhibition are not fully dissociable processes (Van der Ven et al., 2013). This, according to Miyake et al. (2000), is the problem of task impurity: each executive process operates on other processes. Nevertheless, these processes show compelling developmental change in the preschool period and have implications for subsequent achievement.

For the purposes of the present paper, we briefly review findings on the age-related change observed in this period with a focus on executive attention and inhibition. For example, Jones et al. (2003) found evidence of improvements in behavioral inhibition between 3 and 4 years of age on the Simple Simon Task. Children were instructed to follow the command of one large toy animal but not another. Error rates decreased between 3 and 4 years of age and, at age 4, children's response times incremented after making an error whereas this marker of error recognition was not evident in younger children.

On the DCCS task, Zelazo et al. (2003) taught children two sets of rules for sorting a set of cards: one based on shape and one based on color. They found that 3-year-olds understood each set of rules but failed to switch between them. Instead, the first set of rules learned determined the prepotent response on the task. Zelazo et al. (Zelazo and Frye, 1998; Zelazo et al., 2003) interpret this finding as evidence of a failure to reflect on the rules in relation to one another. Other accounts focus on conceptual redescription (Perner and Lang, 2002), latent vs. active memory (Munakata, 2001, 2004), and a failure to disengage attention from a previous rule set (Kirkham and Diamond, 2003). In sum, one can understand the difficulty of 3-year-olds on the DCCS and similar tasks as a problem of thinking about something in two ways simultaneously or, complementarily, as a difficulty of selective attention (Garon et al., 2008). A general finding is that the youngest children perseverate on the first rule pair to which they are exposed. Four- and five-year-olds, in contrast, are significantly more able to resist the prepotent response to the first rule (Zelazo and Jacques, 1996).

In Luria's Tapping Task, children are instructed to tap twice if the experimenter taps once and to tap once if the experimenter taps twice. Like the DCCS, this task requires that children keep both rules in mind simultaneously. In addition, it requires that children inhibit the prepotent tendency to imitate the experimenter. Accuracy on the task improves from 3.5 to 7 years of age (Diamond and Taylor, 1996). Recently, Clark et al. (2013) charted the trajectory of response inhibition and set shifting from 3 to 5 years of age. There was a clear improvement in accuracy on both measures and a reduction in response times.

A different approach, designed to capture individual differences in attention, the Child Attention Network Task (ANT; Rueda et al., 2004), was developed as an extension of the adult flanker task. Colorful fish appear on a screen and the child must “feed” the central fish using the arrow keys on the keyboard. To succeed, the child must focus on the direction that the fish is facing and, in incongruent trials, resist responding based on the orientation of the many other fish (flankers). Reaction time and accuracy improves with age across trial types (congruent, incongruent) and is significantly poorer for incongruent trials. Taken together, the results from these tasks indicate that executive function improves markedly during the period from age 3 to 5 with both qualitative and quantitative change apparent between 4 and 5 years of age.

Executive function, like narrative production, is associated with ToM (Perner and Lang, 1999) and inhibition and working memory are central to this relation (Carlson et al., 2002). Thus, speculatively, relations between executive function and narrative are likely to share variance with ToM through the domain general mechanisms of inhibition and working memory. Also like narrative production, the development of executive function has been associated with development in frontal cortical function (Perner and Lang, 1999). Additionally, improvements in executive function correlate with myelination and branching in the frontal lobe from infancy into middle childhood (Diamond and Taylor, 1996). However, executive function also depends upon a neural network that extends across brain regions. Imaging studies suggest a network that is involved in the resolution of conflict (e.g., between a prepotent and appropriate response) comprised of the anterior cingulate and lateral prefrontal cortex (Fan et al., 2003, 2005) and the inferior frontal and parietal regions (Smith et al., 2004).

Performance on executive function tasks correlates with academic success in mathematics, reading, and writing. Clark et al. (2010) found that children who performed below average on measures of executive planning, attention, and inhibition at age 4 also performed below average on math skills at the first grade level. Interestingly, set shifting did not correlate with any other measure of executive function or with math achievement. Nevertheless, it is clear that set shifting is a central component of executive function. Indeed there has been substantial recent work indicating that set shifting may be an important component of dual language acquisition, supporting the ability to transition between languages and moreover, that dual language acquisition supports precocious development in set shifting (see Kroll et al., 2012, for a review). Although the fields of emergent literacy and executive control receive significant attention individually, relatively little research in typically developing children connects the two fields.

One possibility is that the effects of executive processes may be specific, supporting particular aspects of cognition at particular points in developmental time. Apropos of this hypothesis, Schneider et al. (2006) found that language and working memory at 36 months accounted for significant variance in executive control at 42 and 48 months suggesting that, in early childhood, both factors support subsequent developments in executive function, at least in the short term. However, consistent with the specificity hypothesis, planning, attention, and inhibition did not correlate with working memory and the strength of the prediction from early language to executive control decreased over time. Nevertheless, children with language deficits score significantly more poorly on both verbal and non-verbal executive function tasks than peers without language deficits (Bialystok and Feng, 2009) suggesting that typical language may be important to the development of executive function or, conversely, that typical executive function may be necessary to support language acquisition.

In a large longitudinal study of typically developing children, the National Institute of Child Health and Human Development (2003) found that sustained attention and behavioral inhibition at 54 months partially mediated the relation between home environment and cognitive, school readiness, language, and social outcomes. Other recent work suggests a direct relation between performance on the DCCS and later language and emergent literacy skills such as phonological sensitivity and print awareness (Bierman et al., 2008). In contrast, Coldren (2013) found that whereas DCCS scores correlated with math and district kindergarten exit scores, they did not account for significant variance in reading scores above that accounted for by age and school readiness.

These findings are consistent with the view that the executive processes underlying goal- directed behavior exhibit specificity of prediction: executive processes are not homogeneous but exhibit specific convergent and predictive relations that vary with developmental time. This view is consistent with Garon et al.'s (2008) model integrating unitary and componential approaches to executive function from a developmental perspective (see also Lehto et al., 2003; Huizinga et al., 2006 for alternate integrative models).

A handful of studies have examined the relation between executive processes and narrative production in brain-injured adults. Coelho et al. (1995) found that, in adults with traumatic brain injury (TBI), there was a significant correlation between story structure and executive function such that adults who produced incomplete episodes within the story were also less adept at learning the sorting rule in the Wisconsin Card Sorting Task. Additionally, TBI adults scored significantly lower than average on overall narrative cohesiveness.

In another study, Coehlo (2002) found that individuals with closed head injuries (CHI) produced less coherent episodes and used fewer words overall than adults without head injury and that narrative production was correlated with scores on the Wisconsin Card Sorting Test. Subsequently, Mozeiko et al. (2011) found no differences between a group of adults with TBI and a comparison group on measures of set shifting and inhibition. However, there were significant group differences in narrative organization such that the TBI group's narratives contained fewer content episodes. Further, in the TBI, but not the control, group the correlation between set shifting and story structure was significant. Thus narrative deficits and executive function deficits share variance in adults with closed head as well as traumatic brain injuries. This suggests that the two abilities depend on a shared underlying neural substrate and thus, it is reasonable to expect that executive processes and narrative ability are dependent over developmental time.

Of particular interest in the present research are the convergent and predictive relations between executive processes and narrative production from 4 to 5 years of age. Consider that, in order to tell a good story, a child must engage executive processes. She must maintain the rough structure of the story (what came before and what comes next and how these are related), concentrate on the complete telling of one segment at a time, and nimbly shift between one segment and the next in order to produce a well-structured narrative. In fact, story structure is what makes narrative cohere in a way that facilitates comprehension in a listener (Hudson and Shapiro, 1991; Shapiro and Hudson, 1991). Thus children must organize information in narratives into a set of causal chains that emphasize the temporal sequence and causal relevance of events within the story.

This, in conjunction with the fact that developments in narrative production emerge in concert with developments in executive function, suggests a potential developmental relation between executive processes and the ability to construct narratives. Further, evidence from imaging studies and from brain-injured adults suggests that the neurological networks that support executive function and narrative production are at least partially overlapping and that development in both domains is dependent upon the attention system. What is less clear is the direction of this relation over developmental time. Do executive processes emerge and mature in advance of proficient storytelling or does practice telling stories support the development of executive processes? In a recent review, Diamond (2013) proposed an interdependent model of the relation between active, volitional inhibition and working memory. Successful inhibition requires the contribution of working memory. Similarly, and perhaps not as obviously, working memory requires inhibitory control: focusing the mind and remembering is dependent upon resistance to distraction. Of interest then is the nature of the developmental relation between executive function and narrative development.

The present research focuses on attention, inhibition, and narrative development in early childhood. Because we are interested in the convergent and predictive relations between narrative and executive function, we examine the period between 4 and 5 years of age retesting each participant within a 6 months window to observe how narrative supports executive function and how executive function supports narrative. Although it is possible to assess both executive function and narrative even earlier, we examine this period to minimize floor effects. It is expected that executive processes will correlate within each Wave. Further, we anticipate that each measure (attention, inhibition, and narrative) will correlate across Waves. Of particular interest are the correlations between executive processes and narrative production from Wave 1 to 2.

## Methods

### Participants

**Figure 1:**
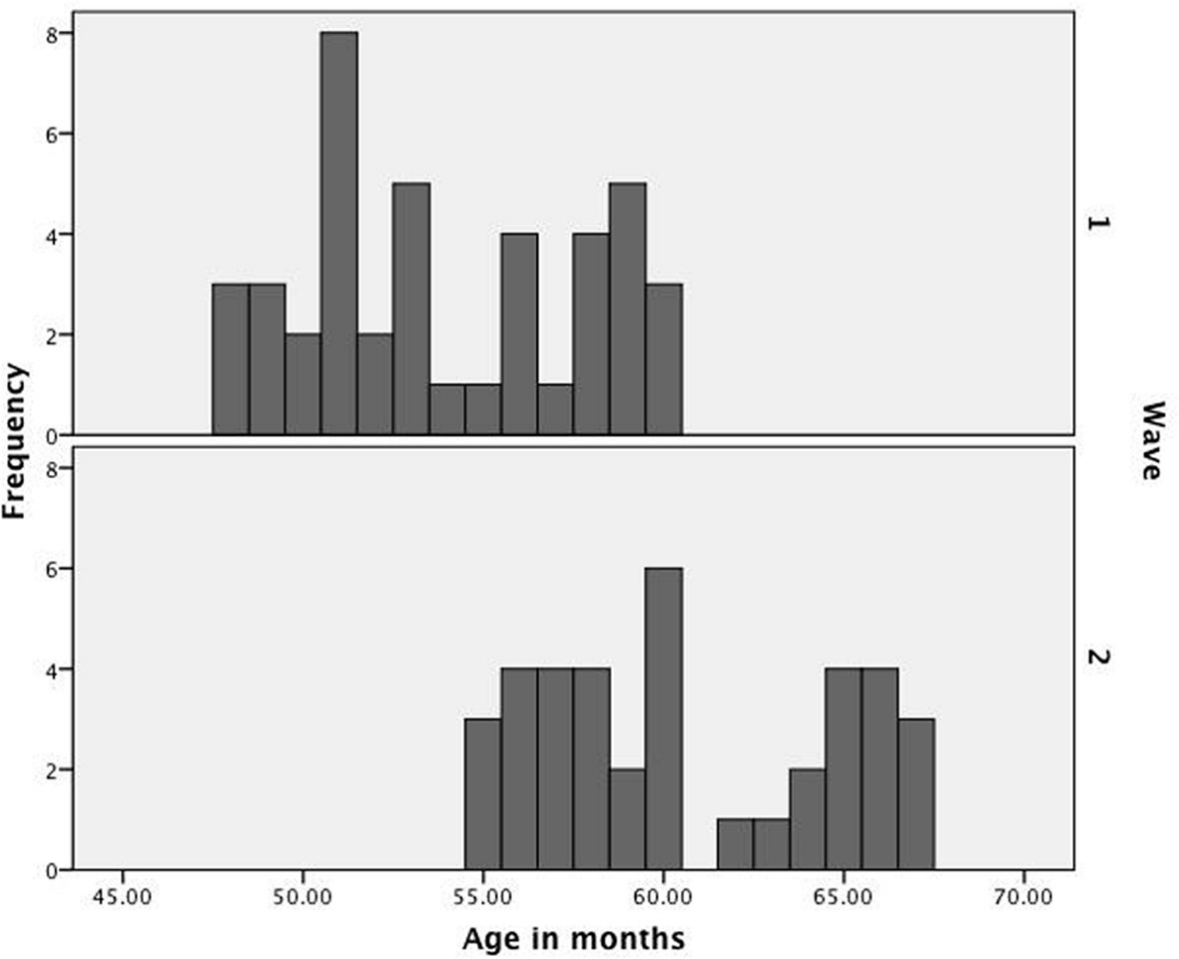
**Distribution of age across Waves 1 and 2**.

A sample of 52 children between the ages of 48 and 60 months (*M* = 53;27) and their primary caregivers participated in the first Wave of this study. Ten children were excluded due to technical difficulties with the audio recorder (7) and general fussiness (3), leaving us with a final sample of 42 children ranging in income from $15,000 to $100,000 per year and with maternal education from 10 to 18 years. All participants were monolingual speakers of American English however roughly one-half reported exposure to a second language, reflecting our presence in a border region. A summary of sample demographics is presented in Table 1. A subset of the sample (38 caregiver-child dyads) returned to participate in the second Wave of the experiment when children were between the ages of 54 and 66 months (*M* = 60;18; see Table 2). Consistent with our primary objective of exploring how skills support one another over developmental time, performance was assessed across Waves using each child as his own control. Because the narrow interval between Waves resulted in some overlap in age (see Figure 1 for a distribution of ages at each Wave), we assess performance on each dependent measure across Waves to insure inter-test interval is developmentally appropriate before proceeding to the cross-lagged analyses.

**Table 1:** **Distribution of selected demographic characteristics of participants Wave 1**.

**Characteristic**	**Boys (***N*** = **16**)**	**Girls (***N*** = **26**)**	**Total (***N*** = **42**)**
**MATERNAL EDUCATION**			
High school or less	–	2 (7.7)	2 (4.8)
some college	4 (25.0)	7 (26.9)	11 (26.2)
college graduate	4 (25.0)	5 (19.2)	9 (21.4)
Post-baccalaureate	8 (50.0)	12 (46.1)	20 (47.6)
**APPROXIMATE INCOME**			
15,000–24,999	–	3 (11.5)	3 (7.1)
25,000–49,999	5 (31.2)	4 (15.4)	9 (21.4)
50,000–74,999	1 (6.2)	5 (19.2)	6 (14.3)
75,000–99,999	5 (31.2)	2 (7.7)	7 (16.7)
100,000–150,000	5 (31.2)	12 (46.1)	17 (40.5)
150,000+	–	–	–
**MATERNAL ETHNICITY**			
Asian	2 (12.5)	–	2 (4.8)
Black/not hispanic	1 (6.2)	2 (7.7)	3 (7.2)
Hispanic	3 (18.7)	9 (34.6)	12 (28.5)
White/not hispanic	10 (62.5)	15 (57.7)	25 (59.5)
mixed race	–	–	–
**SECOND LANGUAGE**			
No	9 (56.2)	11 (42.3)	20 (47.6)
Yes	7 (43.7)	15 (57.7)	22 (52.4)

**Table 2:** **Distribution of selected demographic characteristics of participants Wave 2**.

**Characteristic**	**Boys (***N*** = **14**)**	**Girls (***N*** = **24**)**	**Total (***N*** = **38**)**
**MATERNAL EDUCATION**			
High school or less	–	2 (8.3)	2 (5.3)
Some college	3 (21.4)	6 (25.0)	9 (23.7)
College graduate	4 (28.6)	4 (16.7)	8 (21.0)
Post-baccalaureate	7 (50.0)	12 (50.0)	19 (50.0)
**APPROXIMATE INCOME**			
15,000–24,999	–	1 (4.2)	1 (2.6)
25,000–49,999	5 (35.7)	4 (16.7)	9 (23.7)
50,000–74,999	–	5 (20.8)	5 (13.2)
75,000–99,999	5 (35.7)	2 (8.3)	7 (18.4)
100,000–150,000	4 (28.6)	12 (50.0)	16 (42.1)
150,000+	–	–	–
**MATERNAL ETHNICITY**			
Asian	1 (7.1)	–	1 (2.6)
Black/not hispanic	1 (7.1)	2 (8.3)	3 (7.9)
Hispanic	3 (21.4)	8 (33.3)	11 (29.0)
White/not hispanic	9 (64.4)	14 (58.4)	23 (60.5)
Mixed race	–	–	–
**SECOND LANGUAGE**			
No	8 (57.1)	9 (37.5)	17 (44.7)
Yes	6 (42.9)	15 (62.5)	21 (55.3)

### Measures

#### Narrative elicitation task

“Frog, where are you?” by Mercer Mayer (1969), a 24-page wordless picture book was used to elicit children's narratives. In the story a boy loses his frog and goes on a search to find him. Each page has a single picture of a scene in the story. The book has been used extensively to explore linguistic characteristics of narrative production in children and adults (Berman and Slobin, 1994).

A narrative complexity scale based on Cobo-Lewis et al. (2002) was used to code children's narratives. Previous research indicates that this approach captures aspects of narration that are not confounded with language proficiency in typically developing children. The scale included four subscales, summed to create a Narrative total score. The subscales were: (1) elements (e.g., a story that includes a loss, search, and discovery); (2) sequence (events organized in a causal sequence); (3) syntax (use of verb phrases, conjunctions, and/or adjective clauses); and (4) lexicon (use of a set of words specific to the story). Each subscale was scored separately on a scale from 0 to 12. For the elements subscale, the 12 primary story elements were identified (e.g., the frog is lost, the boy looks for the frog, the boy is sad, etc.). For the elements subscale, one point was assigned for each story element in the narrative. For the sequence subscale, scores were based on the completeness of the causal chain of events. The syntax and lexicon scores reflect a simple count of the number of complex constructions and relevant lexical items in the narrative (up to a total of 12). Inter-rater reliability between the primary coder and the second author was calculated for all of the stories and was >0.81 for Wave 1 and >0.85 for Wave 2.

#### The child attention network test

The Attention Network Test (ANT) assesses the alerting, orienting, and conflict resolution functions of attention in adults and has been adapted for use with children from 4 to 10 years of age (Rueda et al., 2004; Zelazo et al., 2013) and provides a broad measure of the functioning of the attention system. The ANT requires limited verbal instruction consistent with our goal of minimizing potential confounds with language proficiency. A bright yellow fish or a row of five yellow fish appears on the blue screen. The child is asked to help “feed” the central fish by pressing the arrow key corresponding to its orientation. In the neutral condition, only one fish appears. In the congruent condition, five fish appear all facing the same direction. In the incongruent condition, the flanking fish face the opposite direction of the central fish. Prior to presentation of the fish in each trial, one of four cue conditions appears on the screen. In the no cue condition, a fixation cross appears on the screen. In the central cue condition, an asterisk appears where the fixation cross was originally. In the single spatial cue condition, an asterisk appears above or below the fixation cross depending on where the fish will appear. In the double cue condition, two asterisks appear above and below the fixation cross. Resolving the conflict between the target and flanker fish in the incongruent condition has been shown to delay reaction times and activate regions of the lateral prefrontal cortex (Fan et al., 2003). Performance is strongly correlated with both the Block Design subtest of the WPPSI-III, although it is also correlates with the PPVT-IV indicating shared variance with language (Zelazo et al., 2013). Finally, the ANT reliably captures individual differences in attention (Posner et al., 2007).

During the test, the child sat 50 cm from a Dell PC with screen resolution 1280 × 1024. The child placed one finger each on the left and right arrows of the keyboard and used these to indicate the direction of the fish. The test consisted of a practice block of 24 trials, followed by two experimental blocks of 48 trials each. The child received audio feedback through the speakers on the computer. After a correct attempt, the child heard a “Woohoo!” audio-feedback while bubbles flowed from the middle fish's mouth. An incorrect attempt yielded no animation or audio-feedback. Following completion of each block, the child received a sticker as a reward. Reaction times and accuracy were recorded for each of the trials. In the second Wave, a DEX computer equipped with Windows operating system was used for the Child Attention Network Task. For the purposes of the present study, data were collapsed across congruent and incongruent trials to produce two summary scores: ANT Accuracy and ANT Reaction Time (RT).

Luria's Tapping Task. This task has been used to measure response inhibition in children 3½–7 years of age. The child and experimenter sat 45 cm across from each other at a table.

The experimenter held a wooden dowel 30.5 cm in length and 1 cm in diameter. The dowel was passed between child and experimenter to ensure that the child did not tap out of turn. The experimenter instructed the child to tap twice when the experimenter tapped once and to tap once when the experimenter tapped twice. A practice trial was given to insure the child understood the rules. If the practice trial was successful, the child moved on to two sessions of 16 pseudorandom trials each. Response latency and proportion of correct responses were measured. Children's responses were videotaped and coded offline by two experimenters at an inter-rater reliability 0.99.

The two executive function (EF) tasks, the Child ANT and Luria's Tapping Task were chosen from an array of candidate EF tasks for three reasons. First, both measures have been shown to capture changes in EF over the preschool period. Second, together they broadly assess attention itself as well as the known difficulty that children have keeping two things in mind simultaneously and resisting prepotent responses. Finally, both tasks have limited verbal demands thus reducing the potential for confounding performance with language proficiency.

#### Language sample

The child and caregiver were asked to play as they would at home for 10 min with a set of Duplos provided by the experimenter. This session was audiotaped for later transcription. Child language was transcribed into utterance units by one primary transcriber and the second author who completed one-third of the transcripts in common at an inter-rate agreement of 0.80. The transcripts were analyzed using the Systematic Analysis of Language Transcripts software (Miller and Iglesias, 2008). Two summary variables were computed: Number of Unique Words (NW) and Mean Length of Utterance in morphemes (MLU). These measures provide estimates of vocabulary size and grammatical complexity.

### Procedure

This research was approved the Institutional Review Board that oversees the protection of human research participants at San Diego State University. Primary caregivers contacted the lab by phone or email in response to advertisements posted on community-based Internet resources and in local daycare centers. Participants were introduced to the researcher in a 10-min warm-up period in the playroom of the lab while the caregiver filled out a consent form and a demographic questionnaire. All caregivers provided informed consent. Following the warm-up period, participants were taken to an adjacent testing room in the laboratory to complete the Child Attention Network Task (ANT). This room was equipped with a Dell PC on which the ANT program was installed. The child was seated on a chair and used the arrow keys on the keyboard to indicate their responses. The experimenter sat next to the child to explain the task, and behind the child during testing. Following each set of trials, a sticker was given to reward the child. Caregivers observed quietly from across the room throughout EF testing.

Following the Child ANT, the experimenter directed the caregiver and child to a second testing room equipped with a one-way mirror, a Sony Digital Video Camera Recorder Model DCR-TRV 350 in an adjacent room positioned behind the mirror, and a high-quality Audio Technica AT898 Subminiature Cardioid Condenser Lavalier Microphone housed discreetly in a conduit between the two rooms. The microphone recorded onto a Sony TCD-D7 DAT recorder.

For the next 10 min, caregivers engaged in free play with the child with a set of Duplos blocks. Next, the experimenter showed the child the picture book, “Frog, where are you?” by Mercer Mayer (1969), and asked the child to tell her a story using the pictures in the book. Finally, the child and experimenter completed Luria's Tapping Task (Diamond and Taylor, 1996). Tasks were completed in the same order for each participant. It was reasoned that the EF tasks were the most demanding so free play and storytelling were used to break up these tasks to insure compliance and optimal performance. Children also completed a school readiness measure as part of a larger study.

## Results

For each EF task, accuracy and speed were assessed at each Wave. Results from Wave 1 and Wave 2 were analyzed separately and then cross-panel correlations were run to assess the predictive relation from executive function to narrative production and from narrative production to executive function. It was expected that accuracy and speed would correlate across the two EF tasks at each Wave and that narrative production and EF would correlate across Waves.

### Wave 1

Descriptive statistics for the narrative production scores are presented in Table 3. All subscales were normally distributed and the full range of scores was utilized. The inter-item reliability coefficient for narrative production (α = 0.88) was high, indicating good internal consistency. Descriptive statistics for latency and accuracy on the EF measures are presented in Table 4. Total Narrative scores (skew = 0.129, *SE* = 0.365), ANT accuracy (skew = −0.715, *SE* = 0.365), ANT latency (skew = 0.039, *SE* = 0.365) and Tapping accuracy (skew = −0.742, *SE* = 0.365) were normally distributed but Tapping latency exhibited a positive skew (skew = 2.06, *SE* = 0.365). A square root transform was performed on tapping latency scores to normalize the data. Findings were the same for the transformed and untransformed scores therefore we report on the untransformed data. Z-scores were calculated for all dependent measures for the purpose of detecting outliers. Visual inspection of the data revealed no outliers for Narrative or for the EF accuracy measures. A criterion of 2.5 *SD* from the mean was employed based on for the latency measures. Two outliers were identified with reaction times outside this window: 1 participant on the tapping task and 1 on the ANT task.

**Table 3:** **Means, standard deviations, and ranges for narrative production scores at Wave 1**.

**Scale**	***M***	***SD***	**Range**
Elements	6.02	2.32	1.0–11.0
Sequence	5.57	3.08	0–12.0
Syntax	4.76	2.67	0–10.0
Lexicon	7.48	1.70	3.0–11.0
Narrative total	23.83	8.56	7.0–40.0

**Table 4:** **Means, standard deviations, and ranges for tapping task and child ANT at Wave 1**.

**Measure**	***M***	***SD***	**Range**
Tapping accuracy (%)	79.28	17.12	41.0–100.0
Tapping latency (ms)	1817.62	591.10	1130.0–4260.0
ANT accuracy	75.92	14.86	41.1–99.2
ANT latency (ms)	1464.79	290.25	845.0–2159.25

To determine whether age, preschool experience, and language proficiency and exposure influenced performance on narrative and executive function tasks at Wave 1, a MANOVA was conducted with Age, Number of Years in Preschool, and NW and MLU from the Language Sample as covariates, Sex and Second Language Exposure (yes/no) as fixed effects, and ANT latency and accuracy, Tapping latency and accuracy, and Narrative as dependent measures. Power for the full model was high (0.956) and the model was significant, *F*_(5, 32)_ = 5.165, *p* = 0.001, partial η^2^ = 0.450, with an effect of Age, *F*_(5, 32)_ = 3.055, *p* = 0.023, partial η^2^ = 0.330, but no other predictors or covariates reached significance. The analysis repeated with outliers removed yielded no difference in findings. Thus, relations between Narrative and EF cannot be explained by variance due to language proficiency or exposure.

The fact that we did not find a significant relation between the Language Sample and Narrative suggests that our narrative coding system minimized any confound with language proficiency. Narrative storytelling differs from spontaneous language in that storytelling is constrained to a specific subset of lexical items and constructions. Further, in the present study, the Narrative score also reflects the ability to structure language in a causally relevant way that captures all of the salient elements of the story. Thus the total score captures not only words and constructions but also organization and memory. The absence of an effect of language exposure is also not surprising: all participants were monolingual speakers of American English despite some second language exposure. To adequately assess the effects of language exposure, a design including control and comparison groups based upon a fine-grained assessment of the sources and durations of exposure would be necessary.

We proceed with a consideration of the zero-order correlations between EF and Narrative measures as well as the partial correlations controlling for Age. The correlations for the EF tasks and Narrative are presented in Table 5A. As expected, accuracy on the two EF tasks was significantly and positively correlated however when controlling for Age, this relation was not significant. In addition, on the Tapping Task, accuracy was significantly and negatively correlated with latency for both zero-order and partial correlations.

**Table 5A:** **Wave 1 Correlations (outliers included)**.

		**ANT accuracy**	**ANT latency**	**Narrative**	**Tapping accuracy**	**Tapping latency**
ANT accuracy	Zero-order	1	−0.003	**−0.328**	**0.674**	−0.253
	Partial		−0.005	0.010	0.094	0.144
ANT latency	Zero-order		1	0.000	−0.036	0.150
	Partial			−0.178	**0.524**	−**0.238**
Narrative	Zero-order			1	0.133	−0.064
	Partial				0.034	−0.041
Tapping accuracy	Zero-order				1	**−0.446**
	Partial					**−0.244**

Correlations significant at p < 0.05 bolded. N = 42.

A second set of zero-order and partial correlations was computed with outliers on the reaction time measures removed (see Table 5B). These correlations differed in several important ways from correlations based on the full data set. First, ANT accuracy and Tapping latency were moderately related. Second the expected relation emerged between the two latency measures.

**Table 5B:** **Wave 1 correlations (outliers excluded)**.

		**ANT accuracy**	**ANT latency**	**Narrative**	**Tapping accuracy**	**Tapping latency**
ANT accuracy	Zero-order	1	−0.026	−0.009	**0.727**	**−0.420**
	Partial		0.082	−0.153	**0.644**	**−0.351**
ANT latency	Zero-order		1	−0.263	−0.046	**0.416**
	Partial			−0.220	0.072	**0.379**
Narrative	Zero-order			1	0.173	−0.307
	Partial				0.047	−0.246
Tapping accuracy	Zero-order				1	**−0.433**
	Partial					**−0.363**

Correlations significant at p < 0.05 bolded. N = 40.

Third, the negative relation between Narrative scores and ANT accuracy for the zero-order correlations did not replicate when outliers were removed. Finally, the pattern of correlation was consistent across zero-order and partial correlations. That is, controlling for age no longer altered the pattern of results and, as predicted, the accuracy and latency measures were correlated across the two EF tasks.

Contrary to expectations, there was no relation between accuracy and latency on the ANT. This perhaps points to differences in the way that the two EF tasks tap executive processes. In the Tapping Task, which involves both working memory to keep track of the rule and inhibitory control to resist imitating the experimenter, speed may be essential to accurate performance since a delay would place additional demands on working memory. The ANT, in contrast, primarily assesses executive attention: memory demands are limited and responding quickly is less important to performance than maintaining focus on the target.

### Wave 2

For Narrative, all subscales were normally distributed and utilized the full range of scores (see Table 6). The inter-item reliability coefficient was high (α = 0.762), demonstrating good internal consistency and inter-rater reliability was also high (α > 0.85). Total Narrative scores were normally distributed (skew = 0.379, *SE* = 0.393) as were ANT accuracy (skew = −0.790, *SE* = 0.393) and latency (skew = 0.503, *SE* = 0.393). Tapping accuracy exhibited a negative skew whereas Tapping latency exhibited a positive skew (skew = −1.307, *SE* = 0.393, and skew = 1.063, *SE* = 0.393, respectively) and inter-rater reliability for the Tapping Task was high (α = 0.99). A square transform was performed on tapping accuracy and a square root transform on tapping latency to normalize the data. Findings were identical for the transformed and untransformed scores therefore we report on the untransformed data. Table 7 presents descriptive statistics for the latency and accuracy scores for the EF measures. As in Wave 1, Z- scores were calculated for all dependent measures for the purpose of detecting outliers using a criterion of 2.5 *SD* from the mean. One outlier was identified with a Tapping accuracy score outside this window. No outliers were identified on the other measures.

**Table 6:** **Means, standard deviations, and ranges for narrative production scores at Wave 2**.

**Scale**	***M***	***SD***	**Range**
Elements	6.60	1.85	3.0–10.0
Sequence	7.03	2.90	3.0–12.0
Syntax	4.82	2.28	1.0–10.0
Lexicon	8.29	1.27	5.0–11.0
Narrative total	26.76	6.51	15.0–40.0

**Table 7:** **Means, standard deviations, and ranges on tapping task and ANT at Wave 2**.

**Measure**	***M***	***SD***	**Range**
Tapping accuracy (%)	92.44	8.65	69.0–100.0
Tapping latency (ms)	1705.26	490.74	1200.0–3180.0
ANT accuracy	86.57	12.17	57.3–100.0
ANT latency (ms)	1296.32	250.56	798.9–1881.42

To determine whether age, preschool experience, and language proficiency and exposure influenced performance on narrative and executive function tasks at Wave 2, we conducted a MANOVA with Age at Wave 2, Number of Years in Preschool, and NW and MLU from the Language Sample as covariates, Sex and Second Language Exposure (yes/no) as fixed effects, and ANT latency and accuracy, Tapping latency and accuracy, and Narrative as dependent measures. Power for the full model was high (0.924) and, although the model was significant, *F*_(5, 19)_ = 3.0, *p* = 0.037, partial η^2^ = 0.591 there was no effect of any covariate or predictor. The model with the outlier removed was not significant. As at Wave 1, relations between Narrative and EF cannot be explained by variance due to language proficiency or exposure. We now consider the zero-order correlations between EF and Narrative. Removal of the outlier did not alter the pattern of findings and results are reported on the full dataset in Table 8.

**Table 8:** **Wave 2 zero-order correlations**.

	**ANT accuracy**	**ANT latency**	**Narrative**	**Tapping accuracy**	**Tapping latency**
ANT accuracy	1	−0.168	−0.026	**0.437**	**−0.459**
ANT latency		1	−0.297	0.191	0.290
Narrative			1	0.184	−0.203
Tapping accuracy				1	−0.141
Tapping latency					1

Correlations significant at p < 0.05 bolded. N = 38.

As expected, and consistent with Wave 1, accuracy on the two EF tasks was significantly and positively correlated. This, in conjunction with the absence of significant variance attributable to age, suggests that the two EF measures begin to converge in their assessment of executive processes by about 5 years of age. In contrast to Wave 1 however, there was no relation between latency and accuracy on either EF task at 5 years of age, although there was a significant relation between latency on the Tapping Task and accuracy on the ANT. Recall that the only significant relation between accuracy and latency was for the Tapping Task in Wave 1. There was a marginal correlation [*r*_(36)_ = 0.297, *p* = 0.08] between ANT accuracy and Narrative suggesting that the ability to focus attention may be related to narrative production. Of particular interest however, are the cross-lagged correlations from Wave 1 to Wave 2.

### Longitudinal analyses

Before proceeding with the longitudinal analyses, it is important to note that there was 10% attrition from Wave 1 to Wave 2. Included in this attrition were two outliers on the Wave 1 measures. Consequently, these outliers were not part of the sample at Wave 2 and do not contribute data to the longitudinal analyses. Removal of the single outlier at Wave 2 did not alter the pattern of longitudinal findings. Therefore we report all longitudinal findings, including the cross-lagged correlations, on the full sample from Wave 2. We expected each of the measures at Wave 1 to correlate with the same measure at Wave 2. In general, this expectation was supported. Narrative production scores at Wave 1 marginally correlated with narrative production scores at Wave 2. For the EF measures, ANT latency at Wave 1 significantly correlated with ANT latency at Wave 2 and ANT accuracy at Wave 1 significantly correlated with ANT accuracy at Wave 2. Tapping latency at Wave 1 marginally correlated with latency at Wave 2 and Tapping accuracy at Wave 1 significantly correlated with accuracy at Wave 2. The general picture is one of consistency over time in both EF and Narrative.

Next we evaluated the change in performance from Wave 1 to Wave 2 in each dependent measure to determine whether the interval between Waves was sufficient to inform our understanding of development in EF and Narrative (see Table 9). The change in performance was significant for ANT accuracy and latency, Tapping accuracy, and Narrative and marginally significant for Tapping latency. Taken together, the pattern indicates developmental change in individual children in EF and Narrative across a 6-months window in the fifth year. Of particular interest are the cross-lagged relations between EF and Narrative. These were computed with and without outliers and the pattern of findings was comparable. Findings are reported on the full dataset (see Table 10).

**Table 9:** **Change in performance from Wave 1 to Wave 2**.

**Measure**	**Mean difference (Wave 2-Wave 1)**	***SD***	***t*_(35)_**	***p***
ANT accuracy	0.10	0.10	6.05	0.001
ANT latency	−170.19	271.49	−3.76	0.000
Tap accuracy	0.12	0.14	5.15	0.000
Tap latency	−2.48	6.89	−2.10	0.086
Narrative	3.28	9.26	2.12	0.041

With outliers removed, the difference in Tap Latency is significant (p = 0.04) and the difference in Narrative is marginal (p = 0.07).

**Table 10:** **Wave 1–Wave 2 cross-lagged correlations (outliers included)**.

	**W2 ANT accuracy**	**W2 ANT latency**	**W2 narrative**	**W2 tapping accuracy**	**W2 tapping latency**
W1 ANT accuracy	**0.747**	−0.012	**0.337**	**0.632**	−0.216
W1 ANT latency	0.012	**0.476**	−0.060	0.229	0.282^*^
W1 narrative	0.125	−0.012	−0.317^*^	0.010	−**0.379**
w1 tapping accuracy	**0.474**	0.231	0.209	**0.470**	−0.156
W1 tapping latency	−0.216	−0.239	−0.046	−0.224	0.285^*^

Correlations significant at p < 0.05 bolded.

* indicates a marginal correlation at p < 0.10. N = 38.

Narrative at Wave 1 emerged as a significant predictor of Tapping latency at Wave 2, *r*_(36)_ = −0.379, *p* = 0.022, suggesting that practice producing meaningful narratives may support the ability to shift nimbly between responses on a task that taps working memory and inhibition. To explore this finding further, we examined the correlation of each Narrative subscale at Wave 1 with Tapping latency at Wave 2. Both the elements subscale, *r*_(36)_ = −0.374, *p* = 0.025, and the sequence subscale, *r*_(36)_ = −0.387, *p* = 0.02, emerged as significant predictors of subsequent Tapping latency. Importantly both the elements and sequence subscales place demands on working memory and inhibition to recall all of the relevant story elements and to organize them in a meaningful causal sequence. Thus children who are relatively good at constructing a narrative at age 4.5 are likely to be able to shift between arbitrary rules at age 5. There were no other significant relations between Narrative at Wave 1 and EF measures at Wave 2.

Turning to look at the prediction from EF to Narrative, the only significant prediction was from ANT accuracy at Wave 1 to Narrative at Wave 2, *r*_(36)_ = 0.337, *p* = 0.044. The better children were able to focus on the target and resist distraction at Wave 1, the more mature their narratives at Wave 2. We examined the correlation of each Narrative subscale at Wave 2 with ANT accuracy at Wave 1but found no significant effects other than for the total score.

To further clarify the developmental relation between Narrative and EF, partial correlations were calculated from Wave 1 to Wave 2 controlling for the influence of performance at Wave 1 on Wave 2 scores. Narrative production at Wave 1 remained significantly correlated with Tapping latency at Wave 2 even after controlling for Tapping latency at Wave 1 [*r*_(35)_ = −0.380, *p* = 0.020]. In addition, ANT accuracy at Wave 1 remained significantly correlated with Narrative at Wave 2 after controlling for Narrative at Wave 1 [*r*_(35)_ = 0.362, *p* = 0.028].

These results support the notion of bidirectional support between EF and Narrative over developmental time. Focusing and resisting distraction on the ANT in the fourth year predicts the ability to construct a causally coherent narrative in the fifth year, and the ability to construct a narrative in the fourth year predicts the speed with which children can follow arbitrary rules in the fifth year.

One concern was the potential for practice effects from Wave 1 to Wave 2 on narrative elicitation of the frog story. To account for potential practice effects we examined the correlation in narrative production across Waves, controlling for the difference in spontaneous language MLU and NW. The correlation was non-significant, suggesting that narrative production was not subject to practice effects over the 6 month testing interval.

## Discussion

The ability to construct a narrative and components of executive function (e.g., the ability to focus attention, resist distraction, and shift nimbly between arbitrary rules) develop rapidly in the preschool period. Further, these skills are dependent upon overlapping neural substrates, particularly frontal lobe function, and deficits across these skill sets are observed in adults with traumatic and closed head injuries. Lastly, both sets of skills have been implicated in success in the early school years. In spite of these interesting parallels, the relation between narrative and executive function skills during this period has received little attention. The seminal question here is whether these are independent skill sets that just happen to develop concomitantly or whether there is a developmental relation between them such that executive function supports the development of narrative storytelling and practice constructing complex, causally coherent narratives supports development in executive function.

One issue that arises in assessing the relation between executive function and narrative ability is that, although there are many reasons to expect that the two skill sets might be related, causality is difficult to establish. Further complicating this picture is the fact that development can be heterochronous with skills that are deeply conceptually related developing on different timescales. Even though narrative ability and executive function develop across the preschool period, it is not necessarily the case that they do so in lock step. Some aspects of each skill set may develop before others and the relation between skill sets may be such that there is specificity in predictive relations over developmental time. That is, there is no compelling reason to think that all EF measures should equally share variance with the development of storytelling or that relations between EF and narrative should be apparent at any single point in time. For these reasons, we did not necessarily expect to see a relation between executive function and narrative at any one point in time but did anticipate predictive relations in our longitudinal analyses. We begin with a brief review of the primary convergent findings with Waves and then turn to a discussion of our longitudinal findings.

Consistent with our expectations, accuracy on EF measures converged at each Wave. However, contrary to our expectations, the relation between the two measures with regard to speed was much weaker such that we observed a relation between speed and accuracy for the Tapping Task, but not the ANT, in Wave 1 but not in Wave 2. We speculated that speed might be more important in the Tapping Task owing to memory demands. However, it is also the case that, across Waves, Tapping latencies were longer and more variable than ANT latencies and this variability may have contributed to the observed relation between speed and accuracy in Wave 1. At Wave 2, we found no relation between speed and accuracy within EF measures but a significant relation between Tapping latency and ANT accuracy. This effect is somewhat puzzling. This, taken together with the fact that Tapping latency was particularly variable, argues for caution in interpreting this relation between EF measures. With regard to the relation between executive function and narrative, our findings argue against a convergent relation at either 4.5 or 5 years of age. However, predictive relations between the two skill sets emerged in the longitudinal analyses.

Our findings revealed that more advanced narratives at 4.5 years of age were indicative of faster performance on the Tapping Task at 5 years of age. Importantly, this relation was not reciprocal: Tapping latency at 4.5 years of age did not predict narrative ability at age 5. This absence of reciprocity in addition to the fact that there was no convergent relation between Narrative and Tapping at either Wave constrains our interpretation. For example, if it were the case that the two measures correlate due to a third variable such as a shared neural substrate or synchronous developmental timing, we would expect to see convergent relations at each Wave as well as reciprocity in prediction. More likely, given the current evidence, the ability to structure a meaningful, causally coherent narrative supports the subsequent development of speed in responding to arbitrary rules and inhibiting prepotent responses. This finding is similar to recent findings suggesting that bilingualism supports set shifting performance (Soveri et al., 2011). Bilinguals must choose between languages or, put another way, between rule systems and response sets, in every conversation. It is thought that practice shifting between rules and responses underlies a bilingual advantage in executive function, particularly in tasks that involve set shifting. Similarly, we found that the better children were at constructing narratives at 4.5 years, the more quickly they were able to respond to a set of arbitrary rules at 5 years of age. Further, there was suggestive evidence that this relation was driven by children's competence in remembering all of the relevant story elements and organizing them in a causally coherent manner. Thus, skill at keeping track of and organizing key elements in storytelling, like being able to nimbly and appropriately shift between languages, appears to support subsequent speed in responding to arbitrary rules.

We also found a significant positive relationship between children's accuracy on the ANT at Wave 1 and Narrative at Wave 2. Like the relation between Tapping and Narrative, this relation was not reciprocal: Narrative at Wave 1 did not predict ANT accuracy at Wave 2. This finding provides further support for the notion that there are specific relations between narrative production and executive function across developmental time and that these relations reveal the ways in which the two skill sets support one another. This finding suggests that the ability to focus attention and resist distraction at 4.5 years confers benefits in the ability to construct a complex and coherent narrative at 5 years of age. Focusing attention, on what comes first, what comes next, who the relevant players are, and how events are related is key to telling a good story. Similarly, resisting distraction by peripheral information helps a narrator maintain the causal thread that is essential to constructing a meaningful narrative.

Taken together, these findings reveal an asynchronous relationship between executive function and narrative production. Importantly, the nature of this relationship depends upon the specific skills in question and upon the developmental time at which the skills are assessed. We found no strong evidence for a convergence of narrative and executive function skills at either 4.5 or 5 years of age. Rather, specific executive function skills predicted later narrative ability and narrative ability predicted subsequent specific, non-reciprocal, executive function skills. Narrative is not a component of executive function nor is it exclusively an outcome of language development. In fact, we found no relation between spontaneous language and either narrative or executive function. It should be noted, of course, that the particular relations observed between language and narrative will be dependent on the aspects of narrative production that are the focus of the coding scheme. In the present study, we chose an approach that emphasized inclusion of relevant story elements and causal structure as well as more linguistic aspects of storytelling such as syntax and story lexicon. In sum, we found that narrative and executive function are comprised of a set of skills that appear to develop asynchronously during the preschool period and that support subsequent development across skill sets. This finding is consistent with previous research revealing interdependency between executive function and theory of mind (Perner and Lang, 1999; Carlson et al., 2002). However, the present findings extend this work by showing that developments in executive function *per se* do not necessarily precede developments in narrative ability. Rather, there is a true interdependency such that developments in one domain support subsequent developments in the other. This finding is consistent with the work discussed earlier showing a bilingual advantage in some executive function tasks. Further, it extends Perner et al. (2002) approach to the relation between theory of mind and executive function to include the development of narrative ability. Finally, this approach is consistent with Diamond's (2013) interdependent model of the relation between inhibition and working memory and reveals how such an account can conceptually integrate the many aspects of language and cognition that develop rapidly over the preschool period.

It is important to note that we focused the present research on a short window of time late in the fourth year when we expected to see marked development in both narrative and executive function. Our findings are suggestive of intriguing causal connections between these two skill sets. It will be interesting in future research to assess development across the preschool period to clarify these relations. In addition, the present sample size precluded more complex latent variable analyses. Indeed, although the power for the full models in our omnibus tests was high, power for our predictors was not owing to the small sample. These findings require replication with larger samples and modeling approaches to offer more definitive evidence on the relation between narrative and executive function across developmental time.

### Conflict of interest statement

The authors declare that the research was conducted in the absence of any commercial or financial relationships that could be construed as a potential conflict of interest.
